# Delays in initiating rabies post-exposure prophylaxis among dog bite victims in Wakiso and Kampala districts, Uganda

**DOI:** 10.12688/aasopenres.13311.3

**Published:** 2022-12-08

**Authors:** Stevens Kisaka, Fredrick Makumbi, Samuel Majalija, Gloria Bahizi, SM Thumbi

**Affiliations:** 1 University of Nairobi Institute of Tropical and Infectious Diseases, University of Nairobi, Nairobi, 00254, Kenya; 2 School of Public Health, Makerere University, Kampala, 00256, Uganda; 3 College of Veterinary Medicine, Animal Resources and Biosecurity, Makerere University, Kampala, 00256, Uganda; 4 Department of National Disease Control, Ministry of Health, Uganda, Kampala, 00256, Uganda; 5 Paul G Allen School for Global Animal Health, Washington State University, Washington, 001, USA

**Keywords:** delay, dog bite, rabies, post-exposure prophylaxis, Uganda

## Abstract

Background

Although rabies in dog bite patients is preventable through timely initiation of post-exposure prophylaxis (PEP), a number of barriers to achieving PEP exist. This study investigated the delays to initiation of PEP among dog bite patients in the emergency departments of two PEP centers in Uganda.

Methods

A cross-sectional study was conducted among dog-bite patients that presented to two selected rabies PEP centers. A semi-structured questionnaire was used to collect data. Delay to receive PEP was defined as reporting for PEP beyond 24 hours after the bite event. Generalized linear models were used to calculate prevalence ratios and the 95% confidence intervals as a measure of association between delay and patient factors.

Results

Out of 376 participants, just over half (53.5%) were males. The majority of participants (54.0%) were 15 years or older and 28.5% had no formal education. Just over three-quarters (77.9%) had category II dog bite wounds. Nearly 40% delayed to receive PEP, and median (inter quartile range) lag time between bite event and seeking medical care of 18 (41) hours. Compared to education level of secondary or above, patients with no formal education (adj. PR=4.06, 95% CI: 2.69 -  6.10) or primary education (adj.PR=2.15, 95% CI: 1.37 -  3.35), belonging to the lowest socio-economic tertile as compared to the highest (adj.PR=1.58, 95% CI: 1.10 - 2.28), knowing the owner of the biting dog (adj.PR=1.30, 95% CI: 1.02 - 1.65) and having category II wounds (adj.PR=2.31, 95% CI: 1.43 - 3.71) were all associated with delayed presentation for PEP.

Conclusions and recommendations

Delays to receive PEP are common and are associated with poor level of education or low socio-economic status, knowledge of who the dog owner is and less severity of bite wounds. Seeking care irrespective of wound severity or knowledge of dog owner should be promoted.

## Introduction

Rabies remains an important public health problem in Africa and Asia where an estimated 59,000 people die from the disease annually ^1^. Nearly all cases of human rabies are transmitted through bites from rabid dogs, with many of the cases in children 15 years and below ^2^. Rabies is invariably fatal once clinical signs of the disease manifest. However, in humans the incubation period of rabies averages between 15 and 90 days providing an opportunity to prevent clinical rabies following exposure to the virus through bites ^3^. To prevent human deaths from rabies, prompt post-exposure treatment comprising of thorough wound washing, immediate administration of rabies vaccines, and infusion with rabies immunoglobulins (RIG) on the bite wound if indicated is recommended ^4^.

Probable true failures of post-exposure prophylaxis (PEP), where the World Health Organization (WHO) recommendations were followed and patients came down with rabies, have been reported but these are exceedingly rare ^5, 6^. However, deviations from the WHO rabies management recommendations have been widely reported. These include lack of proper wound washing; delays in initiating PEP; not completing the PEP course; and failure to use or improper use of rabies immunoglobulins (RIG) when indicated ^7– 10^. Other factors such as use of vaccines or RIG with low potency may lead to PEP failures, immunocompromised patients and consequent death from rabies among bite patients exposed to the virus ^6, 11, 12^.

Much as rabies has no cure, and by the time of clinical onset it is always fatal, the disease can be prevented even if one has been exposed ^13^. The World Health Organization recommends that immediately after the bite, the wound should be thoroughly flushed and washed for a minimum of 15 minutes with soap and water. In addition, wounds may be disinfected with viricidal agents such as povidone iodine, if available ^14^. Such first aid and pre-clinical procedures have been proven to reduce the chances of progress to rabies by one third ^6, 15^. Clinically, it is recommended anti-rabies vaccine (ARV) is administered and in cases of severe exposure, purified rabies immunoglobulin (RIG) is infiltrated in and around the wound ^14^. The PEP is intended to inactivate the rabies virus if it survived the pre-clinical actions. Studies have shown that timely PEP is nearly 100% effective in preventing development of rabies disease ^15, 16^. It is because of this that the global framework to eliminate human deaths from dog-mediated rabies by 2030 is heavily reliant on ARV use ^17^. So far, over 15 million people receive rabies PEP annually across the world ^14^.

Much as PEP is available, effective and recommended, regions like Africa where rabies is endemic, are still spending the least on PEP ^18^. Besides, there are reports indicating poor implementation of appropriate and prompt PEP for exposed victims, especially in endemic areas like China, India, Iran, Kenya and other countries ^15, 19– 21^. Delays in initiating PEP have been associated with sex, vaccination status of biting animal, whether animal is known or not, type of injury (skin broken or scratched), distance from treatment center, occupation, age, knowledge of rabies, low socioeconomic status, access time of the treatment center and availability of PEP ^19, 22, 23^. These delays often result into a heightened risk of bacterial infection of wounds ^24– 26^ as well as vaccine failure and rabies ^27^.

In Uganda, a recent review of the health surveillance data showed more than 208,000 animal bite injuries and 486 suspected human rabies deaths were reported by health-facilities between 2001 and 2015 ^28^. Several modelling studies in Uganda estimate several hundred cases of human rabies deaths occur ^29^. PEP is free of charge to dog bite patients in public healthcare facilities, after clinicians have assessed the patient needs and eligibility for it. Much as there have been efforts to decentralize this treatment, it is still accessible from only specific healthcare facilities. These facilities receive the vaccine through a ‘push’ system of procurement from Ministry of Health’s National Medical Stores, based on the previous consumption. In addition, these public healthcare facilities also stock the vaccines in their private sections where clients can access them at a fee.

Despite such efforts, suspected human rabies cases continue to be reported in the country ^28^. In addition, undesirable practices like delays to start PEP have been reported ^29^. Much as this information is important for improvement of care and treatment for dog bite victims, there has been minimum effort to study the factors that are likely to be influencing such delays. Previously, we have reported on pre-clinical practices that dog bite patients undertake before seeking medical care ^30^. Here we investigate the compliance to receiving PEP within the first 24 hours and the determinants of delays to initiation of PEP among dog bite patients in rabies-endemic Wakiso and Kampala districts in Uganda.

## Methods

### Study design and area

This study was a cross-sectional survey for all patients that reported to Mulago National Referral Hospital and Entebbe General Referral Hospitals between March 2019 and October 2019, with dog bite injuries. The two health facilities were purposively selected because they were referral hospitals for dog bite PEP services in Kampala and Wakiso districts. These two districts have a high dog population and report the highest annual incidence of animal bites in Uganda ^28^. The average number of dogs per household is two in both districts ^31^. The two districts also have the highest number of dogs vaccinated against rabies in Uganda ^32^.

### Study population and recruitment

The two healthcare facilities were purposively selected based on provision of PEP for dog bites. The sampling unit was a patient with a dog bite injury (DBI). All new patients (i.e. those reporting for initial PEP) were enrolled consecutively as they turned up for PEP. The study participants were adults and children that presented to the healthcare facilities with bite injuries categorized as either II or III as per WHO guidelines ^33^. Patients presenting with category I wounds were excluded because they do not require to receive rabies vaccines.

### Data collection tools and procedure

Data on the outcome and explanatory variables were collected using a pre-tested, coded semi-structured questionnaire. The interviews were conducted either in English or the local language, Luganda, based on respondent’s choice of the language they felt most comfortable expressing themselves. Data on bite event (time, place and dog characteristics), sociodemographic characteristics and other putative risk factors for delays in PEP initiation, were collected. For participants younger than 15 years, the caretaker who accompanied the minor to the treatment center responded to the questions, and where possible, the minor complimented the responses.

### Variables and measures

The dependent variable, time to PEP initiation, was defined as time interval (in hours) from dog bite event to presentation at a health care facility. Time was dichotomized into a new variable “delayed initiation” which was coded as 1: yes, if initiation of treatment occurred beyond 24 hours of the dog bit event, and 0: no, if this occurred within 24 hours. This definition and approach has been used previously in similar studies investigating delays in PEP initiation following bites ^23^.

Data on explanatory variables included sociodemographic factors such as age in years, sex, place of bite event, religion, highest education level attained, employment, marital status and immunization against rabies. Other patient factors included owning of a dog, prior receipt of information about dogs, and perception of severity of the wounds (deep, medium, or superficial). Socioeconomic status (SES) was obtained using a principle components analysis of 11 household items as earlier described in population based surveys ^34^. Household items included possession of radio, television, cell-phone, bicycle, motorcycle, motor vehicle, a piece of land, large farm animals, small farm animals like poultry, a manufactured bed and nature of walls of the house. SES was categorized into tertiles (lowest, middle or highest).

Dog bite injuries were characterized and included bite location on body (lower limb, upper limb, head. torso or combination of other body parts), grade of wound according to WHO classification (category I or II”) and number of wounds (one, two or three and more) ^33^. Data on dog-related factors included rabies vaccination status, knowledge of dog owner or knowing another victim that was bitten by the same dog and perceived health status (sick or not) of the dog. Other factors included distance to healthcare facility (0: within 10km or 1: 1 more than 10km).

### Statistical analysis

Data were double-entered into Epi-info version 7.1.4.0, cleaned and exported to STATA14 (StataCorp.; College Station, TX, USA) for analysis. Descriptive statistics were computed and for continuous statistics included mean (SD) and median (IQR). For categorical variables, frequencies were summarized and tabulated as proportions or percentages. Proportions (percentages) were used to express the magnitude of delays which was disaggregated by independent variables, cross-tabulated and differences in distribution assessed with Persons chi-square test and corresponding p-values. The measure of association between the outcome and explanatory variables was prevalence ratios (PRs) computed using a generalized linear model (GLM) analysis with Poisson family and a log link with robust standard errors. Explanatory variables with p-value < 0.20 or potential confounders were included in the multivariable model. Statistical significance was determined as p≤0.05, and results were reported as PR with corresponding 95% confidence intervals.

### Ethical considerations

The study protocol received ethical clearance from the University of Nairobi - Kenyatta National Hospital Ethics Review Committee (Kenya) REF: P687/09/2018; Mulago National Referral Hospital Research and Ethics Committee (Uganda) REF: MREC 1518; and the Uganda National Council of Science and Technology (Uganda) REF: SS4911. The study procedures were implemented according to approved protocols. Written permission was obtained from hospital directors before commencement of the study. Written informed consent was obtained from participants as well as caretakers of minors prior to the study, while minors provide assent. All data were anonymized and handled confidentially.

## Results

**Figure 1.**
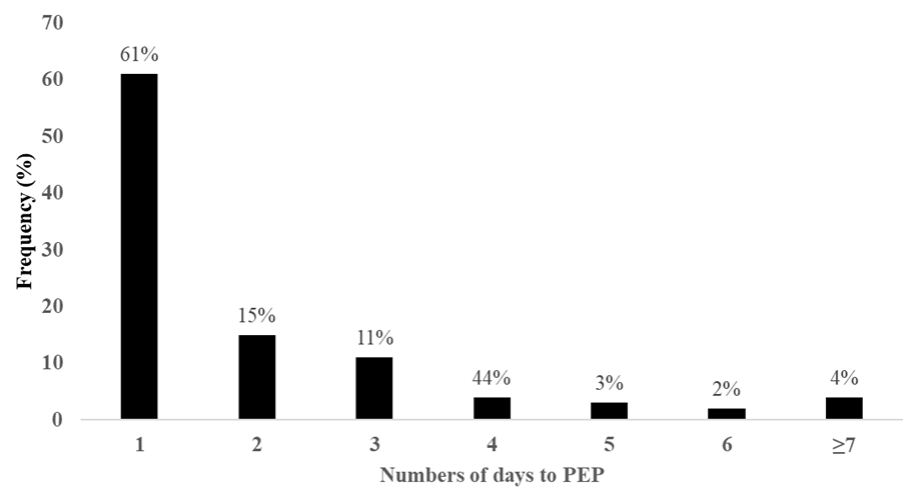
Frequency of delays (number of days) between exposure (dog bite) and PEP.

A total of 376 patients with dog bite injuries were recruited into the study during the study period. Male bite patients, children below 15 years, and participants who were attended to at Mulago National Referral Hospital comprised 54%, 46% and 71% of the study participants respectively. The median distance to any health facility was 11 (IQR, 39.8) kilometers, with patients reporting to Entebbe Hospital and Mulago Hospital travelling a median of 13 (IQR, 18.9) and 11 (IQR, 42.4) kilometers respectively. The median lag between the bite event and presentation to the hospital for PEP was 18 (IQR, 41) hours. We found 40% of the bite patients received rabies vaccines more than 24 hours from the time of the bite, with nearly a quarter of the patients receiving PEP three days after the bite ( Figure 1).

At univariate analysis, delays in PEP were associated with socio-economic status, levels of education attained, knowledge of whether the dog went on to bite someone after inflicting injury on the participant, grade of wound, patient’s perception of the wound severity and number of bite wounds. Other factors including gender, age, place of bite event, employment were not significantly associated with delays in PEP initiation. Table 1 provides the characteristics of the study participants according to whether they had delayed initiation for PEP or not.

**Table 1.** Participant, biting dog and bite injury characteristics for the 376 respondents by delay status.

Variable	Delay present, n (%) N=149 (39.6%)	Did not delay, n (%) N=227 (60.4%)	Total N = 376	p value
Sex				
Male	80 (53.7)	121 (53.3)	201 (53.5)	
Female	69 (46.3)	106 (46.7)	175 (46.5)	0.941
**Age in years**				
≤15	74 (49.7)	99 (43.6)	173 (46.0)	
>15	75 (50.3)	128 (56.4)	203 (54.0)	0.249
**District of bite event**				
Wakiso	77 (51.7)	113 (49.8)	190 (50.5)	
Kampala	72 (48.3)	114 (50.2)	186 (49.5)	0.719
**Religion**				
Christian	119 (79.9)	182 (80.2)	301 (80.1)	
Non-Christian	30 (20.1)	45 (19.8)	75 (19.9)	0.941
Marital status				
Not in union	110 (73.8)	175 (77.1)	285 (75.8)	
In union	39 (26.2)	52 (22.9)	91 (24.2)	0.469
**Highest education level**				
No formal education	31 (13.7)	76 (51.0)	107 (28.5)	
Primary	102 (44.9)	52 (34.9)	154 (40.9)	
Secondary and above	94 (41.4)	21 (14.1)	115 (30.6)	<0.001 *
**Employment status**				
No	78 (52.3)	103 (45.4)	181 (48.1)	
Yes	71 (47.7)	124 (54.6)	195 (51.9)	0.186
Distance to health facility km				
≤10	53(35.6)	85 (37.4)	138 (36.7)	
>10	96 (64.4)	142 (62.6)	238 (63.3)	0.712
Currently own a dog				
No	131 (87.9)	203 (89.3)	334 (88.8)	
Yes	18 (12.1)	24 (10.7)	42 (11.2)	0.650
Immunized against rabies				
No	142 (95.3)	215 (94.7)	357 (94.9)	
Yes	7 (4.7)	12 (5.3)	19 (5.1)	0.799
Get dog information				
No	43 (28.9)	71 (31.3)	114 (30.3)	
Yes	106 (71.1)	156 (68.7)	262 (69.7)	0.618
Socio-economic status				
Lower	24 (16.2)	31 (13.7)	213 (56.7)	
Middle	78 (52.7)	102 (44.9)	73 (19.4)	
Upper	46 (31.1)	94 (41.4)	90 (23.9)	0.03 *
Vaccination of dog against rabies				
No	19 (12.8)	33 (14.5)	52 (13.8)	
Yes	16 (10.7)	31 (13.7)	47 (12.5)	
Don’t know	114 (76.5)	163 (71.8)	277 (73.7)	0.580
Dog looked sick				
No	113 (75.8)	174 (76.7)	287 (76.3)	
Yes	21 (14.1)	25 (11.0)	46 (12.2)	
Don’t know	15 (10.1)	28 (12.3)	43 (11.4)	0.574
Prior dog bit victims by same do				
No	37 (24.8)	67 (29.5)	104 (27.7)	
Yes	40 (26.9)	36 (15.9)	76 (20.2)	
Don’t know	72 (48.3)	124 (54.6)	196 (52.1)	0.034 *
Dog owner known				
No	64 (42.9)	114 (50.2)	178 (47.3)	
Yes	85 (57.1)	113 (49.8)	198 (52.7)	0.167
Category of wound				
II	135 (90.6)	158 (69.6)	293 (77.9)	
III	14 (9.4)	69 (30.4)	83 (22.1)	<0.001 *
Number of wounds				
One	109 (73.2)	130 (57.3)	239 (63.6)	
Two	20 (13.4)	47 (20.7)	67 (17.8)	
Three or more	20 (13.4)	50 (20.0)	70 (18.6)	0.007 *
**Location of bite on body**				
Lower limb	99 (66.4)	140 (61.7)	239 (63.5)	
Upper limb	12 (8.1)	16 (7.1)	28 (7.5)	
Head	8 (5.4)	9 (3.9)	17 (4.5)	
Torso and combination of above	30 (20.1)	62 (27.3)	92 (24.5)	0.435
Perceived wound severity				
Deep	44 (19.4)	21 (14.1)	65 (17.3)	
Medium	63 (27.8)	30 (20.1)	93 (24.7)	
Superficial	120 (52.9)	98 (65.8)	218 (58.0)	0.046 *

*Significance at p≤0.05

On multivariable analysis, we found that the highest level of education attained was a risk factor for delays in presenting for PEP. Holding other factors constant, among the respondents, those with no formal education (adj PR = 4.06, 95% CI: 2.69 - 6.10) and those with primary education (adj PR = 2.15, 95% CI: 1.37 - 3.35) were more likely to delay to seek PEP compared with those who had attained secondary or higher level education. Low socio-economic status was associated with delays in PEP (adj PR = 1.58, 95% CI: 1.10 - 2.28) compared to high socio-economic status. Patients bitten by a dog whose owner is known had higher likelihood of delayed PEP (adj PR = 1.30, 95% CI: 1.02 - 1.65). Additionally, compared with Grade III, patients with Grade II wounds were more inclined to delay receiving PEP (adj PR = 2.31, 95% CI: 1.43 - 3.71). Further, delay in the initiation of anti-rabies PEP was not significantly related to the sex, age group, distance to the healthcare facility, location of the bite on the body, rabies vaccination status of victim or patient perception of severity of wound. In addition, no interaction term between variables was found to be statistically significant. However, much as multiplicity of injuries exhibited statistical significance at bivariable analysis, it turned out not to be significant at multivariable analysis as shown in Table 2.

**Table 2.** Factors associated with delays in seeking post-exposure treatment for the 376 dog bite patients.

Variables	Unadjusted analysis, PR (95% CI)	p-value	adjusted analysis PR (95% CI)	p value
Age				
≤15 years	1.0		1.0	
>15 years	0.86 (0.67, 1.11)	0.250	1.22 (0.91, 1.62)	0.184
Marital status				
Not in union	1.0		1.0	
In union	1.11 (0.84, 1.47)	0.462	1.25 (0.92, 1.71)	0.154
Highest education level				
No formal education	3.89 (2.59, 5.83)	<0.001	4.06 (2.69, 6.10)	<0.001 *
Primary	1.85 (1.18, 2.89)	0.007	2.15 (1.37, 3.35)	0.001 *
Secondary and above	1.0		1.0	
Employment status				
No	1.0		1.0	
Yes	0.85 (0.66, 1.08)	0.187	0.89 (0.69, 1.17)	0.422
Distance to health facility				
≤10km	1.0		1.0	
>10km	1.05 (0.81, 1.36)	0.714	0.99 (0.79, 1.26)	0.991
Immunized against rabies				
No	1.0		1.0	
Yes	0.93 (0.51, 1.69)	0.803	0.803 (0.50, 1.28)	0.359
Get dog information				
No	1.0		1.0	
Yes	1.07 (0.81, 1.41)	0.621	1.19 (0.93, 1.54)	0.157
Socio-economic status				
Lower	1.78 (1.19, 2.62)	0.005	1.58 (1.10, 2.28)	0.014 *
Middle	1.96 (1.27, 3.03)	0.002	1.47 (0.99, 2.16)	0.053
Upper	1.0			
Dog owner known				
No	1.0			
Yes	1.19 (0.93, 1.54)	0.171	1.30 (1.02, 1.65)	0.032 *
Category of wound				
Category II	2.73 (1.67, 4.48)	<0.001	2.31 (1.43, 3.71)	0.001 *
Category III	1.0			
Number of wounds				
One	1.0			
Two	0.65 (0.44, 0.97)	0.034	0.71 (0.51, 1.01)	0.056
Three or more	0.62 (0.42, 0.93)	0.021	0.76 (0.53, 1.08)	0.127
Perceived wound severity				
Deep	1.0			
Medium	0.99 (0.63, 1.58)	0.995	1.06 (0.72, 1.55)	0.764
Superficial	1.39 (0.95, 2.04)	0.090	1.07 (0.76, 1.51)	0.722

^*^Significance at p≤0.05

## Discussion

This study set out to describe the delays in initiating PEP as well as their determinants among dog bite victims presenting for PEP. Timely initiation of PEP is critical in prevention of rabies when patients are exposed to the virus. Consequently, in resource poor settings, human rabies is mainly a result of patients not receiving any PEP, having begun PEP late, or not completing the vaccination schedule ^35– 37^. In this study, where the majority of respondents were in the lowest SES tertile, it was found that nearly 40% of DBI patients reported for initial PEP beyond 24 hours. Such delays were associated with the respondent having attained less levels of formal education; knowing the owner of the biting dog; and the degree of severity of the bite wound.

In the study sample, 56.7% of the respondents were in the lowest tertile of socioeconomic status (SES). This finding agrees to the inverse relationship between SES and health events which generally exists. With regard to dog bites, this socio-economic gradient has been described in various studies leading dog bites to be considered as majorly a problem of poor and vulnerable populations and rabies as a neglected disease of poverty ^38, 39^. Such an observation may be due to dogs living in lower income settings not being afforded the care and management that is relevant in minimizing bite risks. Nonetheless, other authors have attributed the occurrence of dog bites in low income areas to large numbers of children playing outdoors, few homes with adequate fencing, poor dog control, and a high proportion of large-breed dogs owned for protective purposes ^38^.

In our study, 149 (39.6%) respondents presented for PEP beyond 24 hours. In addition, the median time to presentation was 18 hours which is comparable to that in an earlier study ^29^. Further, our findings are comparable to those in China ^15^, far higher than in Bhutan but lower than in India ^40, 41^. However, the lower delay among animal bite patients in Iran may be explained by the authors defining the delay as presentation beyond 48 hours contrary to that of this study. Nonetheless, the variability of delays is indicative of the different health seeking behaviors that are prevalent in different socio-cultural settings. 

In our study, respondents with no formal education and those with primary education were more likely to delay to seek PEP compared with those who had attained secondary or higher level education. A number of authors on rabies have identified education achievement levels a major predictor for knowledge about the disease. There is evidence for lower education levels to predict a low level of knowledge ^15, 42^. An individual that is not educated may find it difficult to access and interpret information about the prevention and management strategies of dog bite wounds. Consequently, they may delay in seeking PEP. This means that much as health education drives might be of importance in the study area as regards management of dog bites, particular emphasis is needed for those with primary education and less. They may require health education materials that are packaged in simpler ways to meet their needs.

Patients in the lowest SES tertile were 1.6 times more likely to delay to present for PEP compared to those in the upper tertile. This might be as a result of the perceived and real costs that are incurred in transport to the healthcare facilities as well as treatment itself. In a similar setting, Ethiopia, a study showed that of the total costs related to post-exposure treatment, non-health related expenses (mainly travel and time) made up to 70% of the total cost ^43^. Several other studies have found socio-economic status to be a major factor associated with PEP seeking behavior by the victims following potential rabies exposures ^40, 43, 44^.

Further, patients bitten by a dog whose owner is known had a tendency of delaying to PEP. This finding may be explained by the wrong perception among people that domestic and known animals are less risky in spreading rabies compared to freely roaming ones ^15^. In addition, it is instinctive that victims bitten by dogs of unknown ownership will most likely seek care immediately. This is because unknown ownership comes with unknown history and health status of the biting dog. Besides, if the owner is known, the victims can easily establish the status of those factors and become contented of a lesser risk thus delaying to present for PEP. Much as there are risks with this approach, it may be the plausible reason for this study finding.

In this study, we found that severity of wound was an influential factor associated with delay in seeking PEP Victims with grade II wounds were over twice more likely to present beyond 24 hours compared with those having grade III bites. It should be noted that grade III wounds present as bites that penetrate the skin and draw blood hence being more serious than grade II. Because of appearing less serious, a victim with grade II wound (s) may take their time to appreciate the risks involved. Therefore, a bite that appears harmless on the surface may explain the longer time taken to seek PEP. Our findings are in agreement with other authors who have found that people who have deeper wounds will visit health centers as soon as possible to receive anti-rabies treatment care ^22, 43^.

Contrary to what we expected, age, employment status and distance to the PEP facility were not associated with presentation beyond 24 hours. In addition, the cross sectional design of the study limits our ability to draw firm conclusions on the potential causal-effect relationship. Secondly, the data were collected mainly through self-reports which are prone to recall bias. Efforts to mitigate this were through verification of responses where possible. Lastly, the study was hospital-based with a convenience sample hence the findings should be interpreted within this context.

## Conclusions

This research shows that dog bite patterns in Uganda are similar across key characteristics for example age, gender and employment status. Additionally, this study provides information on determinants of delays to seek PEP. More than a third of rabies exposure victims presented to the PEP centers beyond 24 hours after the bite event despite the PEP being freely available. The likelihood of late presentation following rabies exposure was greater among those who are lowly educated, those in low SES categories, those bitten by dogs of known owners and those that had less severe bite wounds. We recommend tailored health education programs for the identified vulnerable groups of people. Secondly, there is need to highlight the elevated rabies risk to patients bitten by dogs whose owners they know and those with wounds that may appear less serious.

## Data Availability

Data for the study cannot be shared publicly because the data contains potentially identifying information. The restriction has been imposed by the Mulago National Referral Hospital Research and Ethics Committee, Uganda, (MREC), an IRB that approved the study. Data are available from MREC (Email: nakwagala@yahoo.com) for researchers who meet the criteria for access to confidential data.
