# Supplementary material for: Delays in initiating rabies post-exposure prophylaxis among dog bite victims in Wakiso and Kampala districts, Uganda
Source: AAS Open Res. 2022 Dec 8;4:49. Originally published 2021 Oct 15. [Version 3] doi: 10.12688/aasopenres.13311.3 (PMC9648361; doi:10.12688/aasopenres.13311.3)
Supplement: Peer review history

# Peer review: Reviewer response for version 2

**DOI:** 10.21956/aasopenres.14464.r29359

**Published:** 2022-10-31

**Authors:** Claude T. Sabeta

**Affiliations:** 1 Rabies Reference Laboratory, Agricultural Research Council-Onderstepoort Veterinary Institute, World Organisation for Animal Health (WOAH), Pretoria, South Africa; 2 University of Pretoria, Pretoria, South Africa

Kisaka *et al. *investigated the factors resulting in delays in initiating PEP among dog bite victims in two PEP facilities in Wakiso and Kampala districts (Uganda). This is a well written study and important to identify specific intervention points for dog bite victims. 

 Just three minor issues:

1. On page 9/14, correct the word "Ina".
2. Please clarify what medical staff assess patient needs and eligibility?
3. What is the risk of dog rabies in these two districts? Are any dog specimens submitted for rabies testing?

Is the work clearly and accurately presented and does it cite the current literature?

Yes

If applicable, is the statistical analysis and its interpretation appropriate?

Yes

Are all the source data underlying the results available to ensure full reproducibility?

Yes

Is the study design appropriate and is the work technically sound?

Yes

Are the conclusions drawn adequately supported by the results?

Yes

Are sufficient details of methods and analysis provided to allow replication by others?

Yes

Reviewer Expertise:

rabies epidemiology and diagnosis

I confirm that I have read this submission and believe that I have an appropriate level of expertise to confirm that it is of an acceptable scientific standard.

# Peer review: Reviewer response for version 2

**DOI:** 10.21956/aasopenres.14464.r29011

**Published:** 2021-11-30

**Authors:** Enock Madalitso Chisati

**Affiliations:** 1 Department of Rehabilitation Sciences, Kamuzu University of Health Sciences (KUHeS), Blantyre, Malawi

All comments have been appropriately addressed.

Is the work clearly and accurately presented and does it cite the current literature?

Yes

If applicable, is the statistical analysis and its interpretation appropriate?

Yes

Are all the source data underlying the results available to ensure full reproducibility?

Yes

Is the study design appropriate and is the work technically sound?

Yes

Are the conclusions drawn adequately supported by the results?

Yes

Are sufficient details of methods and analysis provided to allow replication by others?

Partly

Reviewer Expertise:

Exercise and Health, Public Health

I confirm that I have read this submission and believe that I have an appropriate level of expertise to confirm that it is of an acceptable scientific standard.

# Peer review: Reviewer response for version 1

**DOI:** 10.21956/aasopenres.14438.r28966

**Published:** 2021-10-27

**Authors:** Enock Madalitso Chisati

**Affiliations:** 1 Department of Rehabilitation Sciences, Kamuzu University of Health Sciences (KUHeS), Blantyre, Malawi

The article investigates factors that lead to delays in accessing PEP among individuals with dog bites in selected healthcare facilities in Uganda.

 The authors should be commended for a sound and important article that has covered almost all requirements. I have very few comments that need to be addressed by the authors. Once these are addressed I would recommend this article for indexing.

**Comments:**

- Paragraph 2: Spell out abbreviations when first used. Abbreviations such as PEP as used in paragraph 2.

**Methods:**

**Study population and recruitment:**

- This section has only provided information on study population and not how the participants were recruited. Can the information on how participants were recruited be provided as well?

**Discussion:**

- The first paragraph should present the main findings of the study. The other paragraphs following can then discuss other findings found which support the main findings.

Is the work clearly and accurately presented and does it cite the current literature?

Yes

If applicable, is the statistical analysis and its interpretation appropriate?

Yes

Are all the source data underlying the results available to ensure full reproducibility?

Yes

Is the study design appropriate and is the work technically sound?

Yes

Are the conclusions drawn adequately supported by the results?

Yes

Are sufficient details of methods and analysis provided to allow replication by others?

Partly

Reviewer Expertise:

Exercise and Health, Public Health

I confirm that I have read this submission and believe that I have an appropriate level of expertise to confirm that it is of an acceptable scientific standard.

# Response

**Published:** 2021-11-23

**Authors:** Stevens Kisaka

**Affiliations:** School of Public Health, Makerere University, Uganda

**Comment 1:**

**Paragraph 2: **Spell out abbreviations when first used. Abbreviations such as PEP as used in paragraph 2.

**Response: **This has been done.

**Comment 2:**

**Methods: Study population and recruitment:**

- This section has only provided information on study population and not how the participants were recruited. Can the information on how participants were recruited be provided as well?

**Response: **The information on recruitment of respondents has been provided under the section.

**Comment 3:**

**Discussion:**

- The first paragraph should present the main findings of the study. The other paragraphs following can then discuss other findings found which support the main findings.

**Response: **The first paragraph has been improved by incorporating the key findings.
